# Functional Alkali Metal-Based Ternary Chalcogenides: Design, Properties, and Opportunities†

**DOI:** 10.1021/acs.chemmater.3c01652

**Published:** 2023-09-22

**Authors:** Hannah McKeever, Niraj Nitish Patil, Manoj Palabathuni, Shalini Singh

**Affiliations:** Department of Chemical Sciences and Bernal Institute, University of Limerick, V94 T9PX Limerick, Ireland

## Abstract

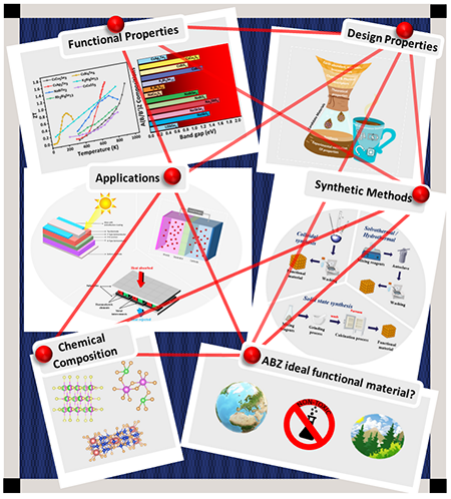

The search for novel materials has recently brought research attention to alkali metal-based chalcogenides (ABZ) as a new class of semiconducting inorganic materials. Various theoretical and computational studies have highlighted many compositions of this class as ideal functional materials for application in energy conversion and storage devices. This Perspective discusses the expansive compositional landscape of ABZ compositions that inherently gives a wide spectrum of properties with great potential for application. In the present paper, we examine the technique of synthesizing this particular class of materials and explore their potential for compositional engineering in order to manipulate key functional properties. This study presents the notable findings that have been documented thus far in addition to outlining the potential avenues for implementation and the associated challenges they present. By fulfilling the sustainability requirements of being relativity earth-abundant, environmentally benign, and biocompatible, we anticipate a promising future for alkali metal chalcogenides. Through this Perspective, we aim to inspire continued research on this emerging class of materials, thereby enabling forthcoming breakthroughs in the realms of photovoltaics, thermoelectrics, and energy storage.

## 1 Introduction

There is an ever-increasing demand for advanced functional materials to meet the needs of the evolving technologies and industries. The quest for discovering potential technologically relevant materials with exciting properties becomes very important when considering the global energy crisis, where shortages of oil, gas, and electricity are being experienced across the globe. Countries are aiming to combat energy shortages by facilitating the transition from fossil fuels toward renewable, efficient, and climate-neutral energy. This has promoted a faster transition to cleaner energy within research fields in the form of energy conversion and storage devices. Energy conversion and storage technologies require new materials with new relevant functionalities that can enable substantial performance improvement of the current state-of-the-art technologies.

As described previously by Prof. Alex Zunger, materials can be defined by their constituting atoms (as determined by atomic number), composition (ratio of elements), and structure (crystal, nano- and microstructures, short order or long order).^1^ Different materials, even with the slightest difference in their constitution, composition, and arrangement, will have different mechanical, physical, electronic, or optical properties. These factors can be regarded as the genetic code of the organic and inorganic compounds, upon which the final properties of the material are highly dependent. The properties desirable for a particular device are always well-known; however, the exact “atoms–composition–structure” sets of materials with such properties are difficult to identify. From penicillin to fullerenes, there are several examples of accidental material discoveries with fascinating properties. Nevertheless, the relentless growth of technologies of the present day demands a systematic approach to material discovery. This is where material design essentially becomes a collaboration of materials science, solid-state chemistry and physics, and computational theoretical research.

A specific example of successful material discovery of the past decade is the lead halide perovskite.^2,3^ Perovskites exploded onto the research scene by exhibiting high charge carrier mobilities and high absorption coefficients with tunable band gaps, alongside the advantages of low-temperature and facile processing.^4−6^ Here, experimental and theoretical research have contributed to demonstrate astonishing escalations in the power conversion efficiency of perovskite-based photovoltaics. With an initial efficiency of ∼4% in 2009, the perovskite solar cell reached >25% efficiency by the end of 2020.^7^ Unfortunately, their great prospects are accompanied by two serious limitations: air instability and toxicity. Perovskites materials dissociate rapidly in the presence of moisture; this degradation also poses harm to the environment, as it creates the opportunity for lead, a carcinogen, to leach into the ecosystem. While these limitations are intrinsic, with the uncertainty of being resolved, they hold the key to the future of energy-conversion material design.

**Figure 1:**
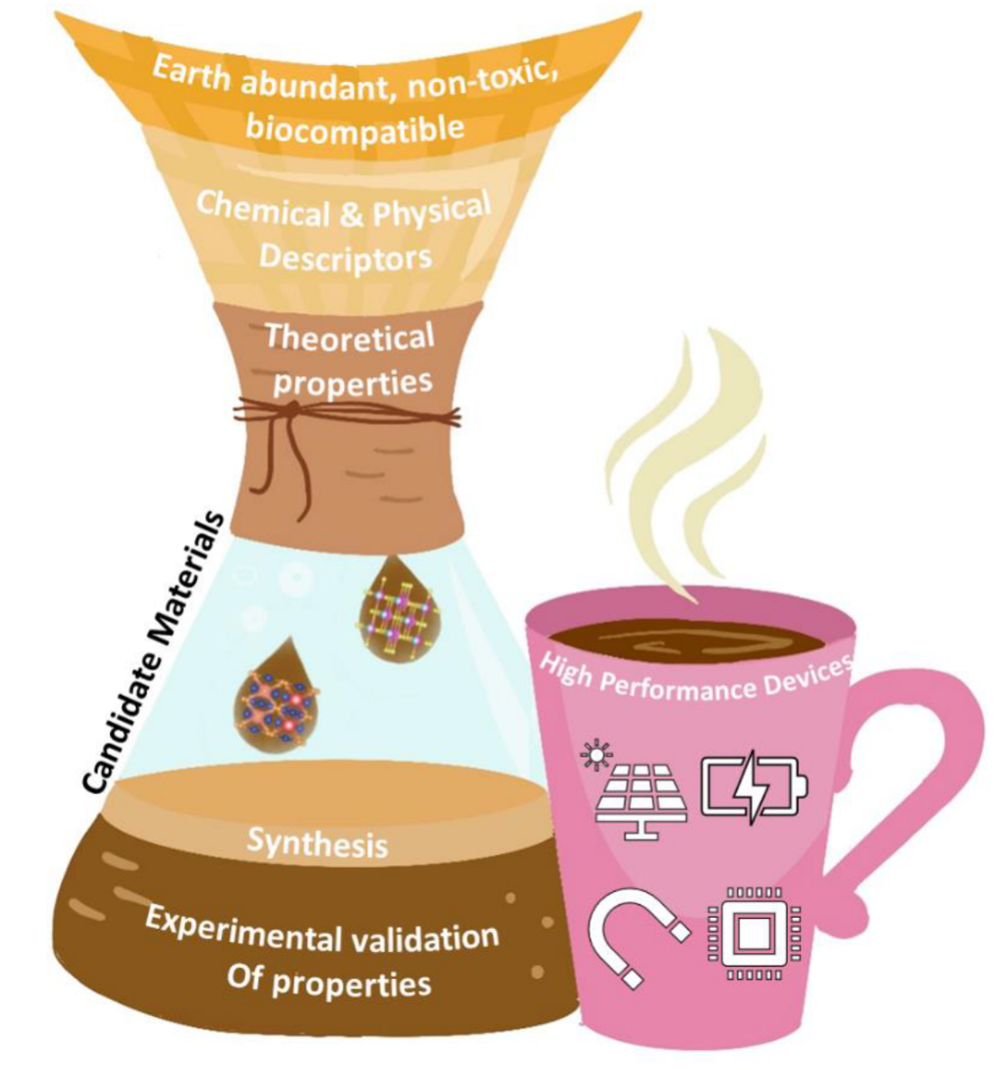
Schematic illustration of the inverse material design “filtered” approach. Each layer of the coffee filter funnels down the possible candidate materials to be synthesized, followed by validation of their properties before finally making high performance devices in the form of the finished cup of coffee.

When looking to the future of such materials, the fascinating properties of materials are no longer enough to warrant their use. The future is dependent on sustainability, a lesson learned from the diminishing level of fossil fuels. If perovskites can be mimicked in structure and properties but with sustainable, nontoxic materials, it could be possible to create the next generation of energy conversion devices. This idea has triggered a new field of research in “perovskite-inspired” materials (PIMs).^8,9^ The strategies to identify the new classes of PIMs have been based on searching for chemical analogs of perovskite materials and structures using an “inverse design approach” (Figure 1). Here, the high-throughput screening via a layered, “filtered” approach gives a promising direction to competently analyze the raw chemical landscape and conduct the systematic discovery of hitherto missing yet realizable PIMs. A topical review by Huang et al described the different material classes of PIMs, including Sn/Ge perovskites, binary halides, chalcogenide perovskites, alkali metal-based chalcogenides, etc.^10^ Among these, alkali metal-based chalcogenide is one of few classes of materials to provide hope for energy materials by ticking the boxes of abundance, stability, and biocompatibility while showing theoretical and experimental potential for high performance. The family of alkali metal-based chalcogenide materials includes compositions with 8 and 18 valence electrons per formula unit. Because of the closed-shell s^2^p^6^ and s^2^p^6^d^10^ electron count, many of these materials are semiconductors with optoelectronic, thermoelectric, piezoelectric, ferroelectric, and other interesting and useful properties. Intriguingly, in these chalcogenide families, varying the metal to chalcogen ratios can lead to the formation of different structures that can have different crystal phases and band gaps and hence different optoelectronic properties.

**Figure 2:**
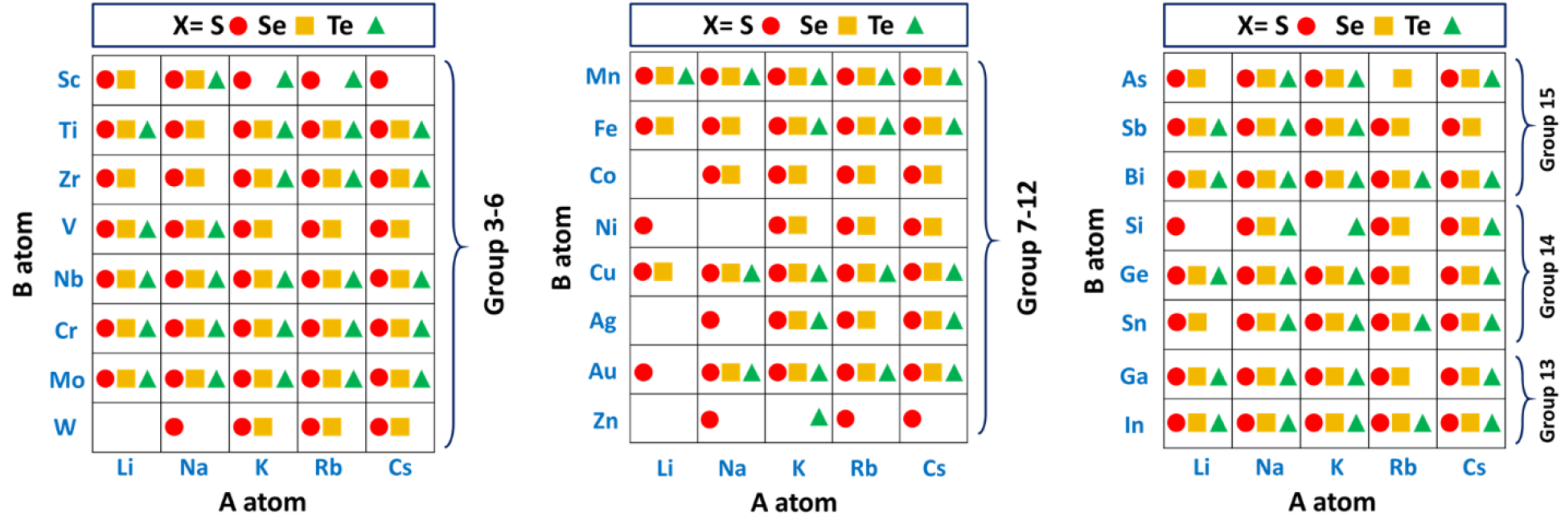
Library of ABZ compounds. The group Z elements are shown as red circles (sulfides), yellow squares (selenides), and green triangles (tellurides)].

In our group, the research is focused on functional nanomaterial design based on metal chalcogenides for energy conversion and storage applications.^11−31^ In our current search for stable and synthesizable multielement inorganic nanostructures (based on alkali metal chalcogenides that exhibit interesting optoelectronic properties), we limit the material composition to ternary systems. Multinary chalcogenides provide unlimited control with which one can influence the composition-dependent parameters (e.g., crystal structure, particle dimensions, and electronic structure) and can simultaneously complicate the search for conceivable materials. Hence, ternary compositions become the conscientious choice for understanding the composition–structure–function relationships without adding extra constraints in the design and synthesis process. Given this, we focus on ternary chalcogenides composed of alkali metal cations, chalcogen anions, and a third cationic element. Here we define the alkali metal-based chalcogenides class as ABZ where A = Li, Na, K, Cs, Rb, and Cs, which have stable +1 charge states. The B elements include metals such as Bi, Sb, In, Ga, Fe, Cu, Ag and Au, and Z-site anions are S, Se, and Te (Figure 2). We are defining this class of materials as “alkali metal based ternary chalcogenides”, but we do not include all metals as possibilities for the B cation. We have categorized the B element based on favorable theoretical predictions with the added advantage of the elements being relatively abundant and benign. Figure 2 visually displays the compositions of ABZ that have already been synthetically achieved in the literature. The three tables in the figure are divided into the groups from the periodic table in which the B elements belong. While many materials in this class have reported synthesis methods, a lot of them have not been further researched in terms of characterization of properties and variations in phases and compositions, thus leaving a wide scope for future research on the ABZ class.

While we have seen materials with other B metals (not shown in Figure 2) show attractive properties in the past, we believe it is worth focusing research on compositions that already fulfill the sustainability and biocompatibility requirements. There is no denying the impact perovskites have had on this field of research and on expanding the knowledge of material–property relationships for energy conversion devices. However, we believe it is time to focus research in a new sustainable direction from the point of view of the material chemists who work in retrograde using an inverse design approach.

In this Perspective, we will shed light on alkali metal-based chalcogenides (ABZ) as the next generation of energy conversion materials (the compositions can be seen in Figure 2). The variations in crystal structures and compositions of these perovskite-inspired materials are explored to highlight their properties. Subsequently, we delve into the possibility of further engineering of compositions to enhance properties through substitution with other metals. We explore the synthesis methods for ABZ and their importance, and we showcase the opportunities for application of these materials. Finally, the need for future research and the challenges being faced in this area are also considered to bridge the gap from lab to fab.

## 2 Stoichiometry and Structures

**Figure 3:**
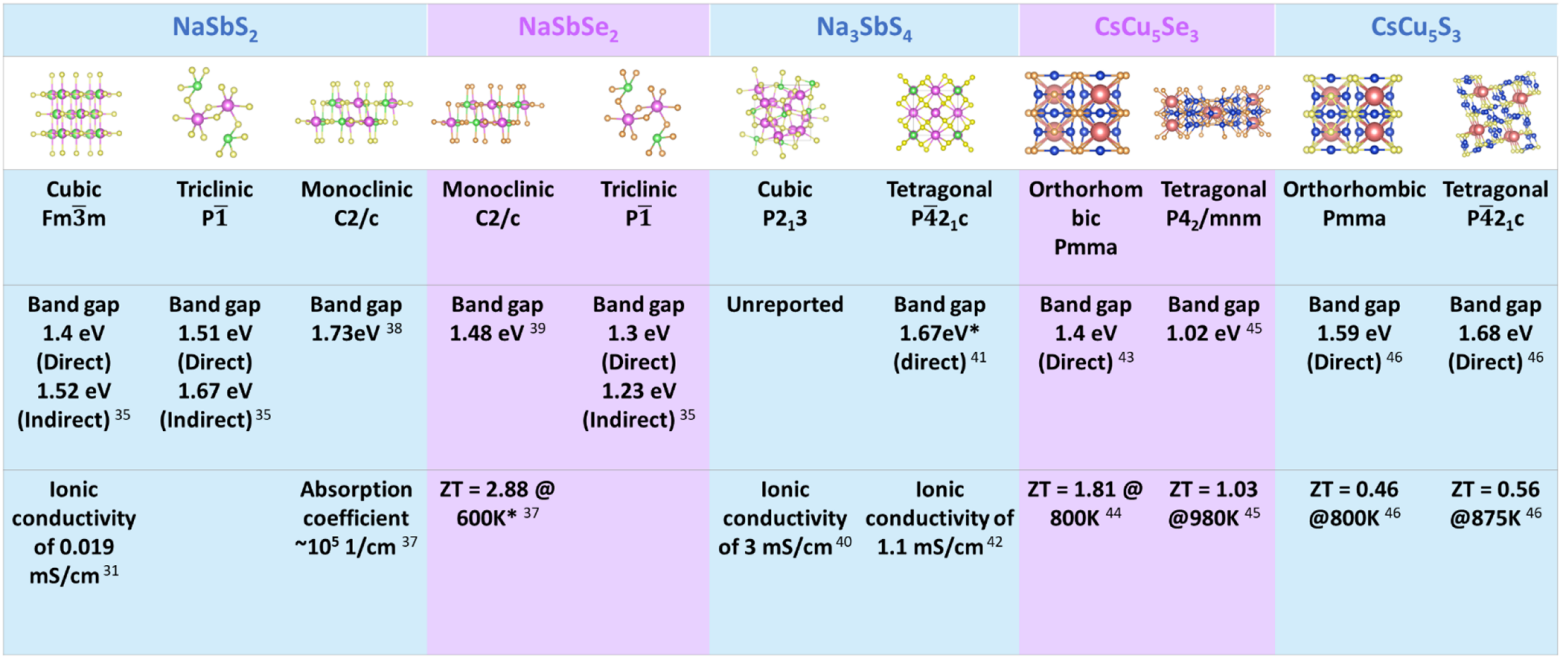
Crystal structures and phases of ABZ materials with basic functional properties.^31,35−46^ An asterisk (*) denotes the theoretically calculated values.

Metal chalcogenides are a class of materials that have seen a plenitude of research due to their attractive properties and their facile solution-phase synthesis methods. Starting from the prototypical II–VI metal chalcogenides (e.g., CdSe) to the layered transition metal chalcogenides (e.g., MoS_2_ and WSe_2_), they exist in a variety of crystal structures and therefore appealing physiochemical properties.^32^ The addition of alkali metals as additional elements to a binary metal chalcogenide to make ABZ structures introduces a broad landscape of optoelectronic properties that changes with the atoms, composition, and arrangements of compounds.^33,34^ A facile way to vary material properties and broaden the prospects is through the makeup of the material itself. Examples of properties that can be varied are the band gap and the figure of merit ZT, which can be expressed by  where *S*, σ, *T*, and κ represent the Seebeck coefficient, electrical conductivity, absolute temperature, and thermal conductivity, respectively. For instance, addition of the alkali metal cesium to copper sulfide and copper selenide can vary band gap and ZT as shown in Figure 3, with CsCu_5_Z_3_ showing a broad range of ZT values and direct band gaps in the range of 1.4–1.59 eV, which is in the ideal range for PV materials. Figure 3 depicts a select range of properties the materials in this class have, properties that are governed by their atomic make up, crystal phase, and crystal structure. The ABZ class spans over a vast number of materials, giving hope in the search for materials to advance energy conversion and stage technologies.

Herein we discuss the excellent structural flexibility of ABZ and how this creates an expanisive composition space of materials by varying (1) different polymorphs of the same elemental composition and (2) different crystal structures with variations of elemental ratios. These factors create a space with an infinite number of materials to utilize.

### 2.1 Polymorphism

The ABZ material properties can also be adapted through polymorphism, where the same compounds have different atomic arrangements of their unit cell and, therefore, belong to different crystal systems. The class of alkali metal chalcogenides exhibits both 3D and layered crystal structures, expanding the field of possible properties and therefore applications of alkali metal chalcogenide materials. Figure 3 displays a few ABZ material sets that exist in different polymorphs. Each polymorph varies from another in terms of its band gap, conductivity, and properties, which are significant several energy conversion and storage devices. For instance, the orthorhombic and tetragonal polymorphs of CsCu_5_S_3_ are prime examples of how crystal structures play a role in material properties and how even the smallest structural changes have effects.^46^ In both polymorphs of CsCu_5_S_3_, the structures consist of columns of Cu_4_S_4_ as the building blocks, and the resulting orthorhombic or tetragonal structure depends on if the building blocks are stacked side by side to create a 2D material or at four apexes to form a 3D network. This emphasizes why detailed studies need to be carried out on these materials to study different possible crystal structures of materials and harness the best properties from them. This also showcases how there are an infinite number of possibilities for materials within the ABZ class that have yet to be reported or researched in detail. Another example can be seen from Figure 3, where NaSbS_2_ and NaSbSe_2_ both have two polymorphs each. NaSbS_2_ exists in cubic and triclinic systems, while NaSbSe_2_ exists in the monoclinic and triclinicsystems, although the atomic ratios remain in the ABZ_2_ format.^35,39^ The polymorphism in NaSbS_2_ can be induced during synthesis varying the reaction temperature. The cubic crystal system is achieved below 300 °C, and the triclinic system is achieved above 300 °C.^35^ Na_3_SbS_4_ also displays similar behavior, with the metastable tetragonal phase dominating below 120 °C, which transitions to the thermodynamically stable cubic phase above this temperature.^42^ The temperature-dependent phase transitions within the ABZ class are a phenomenon that is unseen when using high temperature/solid state techniques for materializing this class, signifying the importance of nonconventional synthesis approaches to synthesize and stabilize the metastable crystal structures.

The arrangement of atoms in a crystal lattice plays a dominant role in dictating the optoelectronic and ionic conductivity properties of materials. In a simple example, the monoclinic phase of NaSbSe_2_ has a wider band gap than its triclinic polymorph due to the change in band structure, which comes with a change in crystal structure.^35,40^ In the case of ionic conductivity, changes in the atomic arrangement govern the energy barrier for ion diffusion channels in the lattice. Cubic Na_3_SbS_4_ has a high degree of symmetry, giving Na ions a 3D diffusion pathway; this ion pathway creates a small gap of 2.85 Å for Na ions to move across in comparison to the tetragonal polymorph, in which the gap is 4.83 Å.^40^ The energy gap is directly associated with the material’s activation energy, which results in a high diffusion coefficient and therefore high ionic conductivity. Hence, the change in crystal structure from tetragonal to cubic boosts material properties by twofold, thus making Na_3_SbS_4_ a candidate for all-solid-state battery electrolytes.

### 2.2 Stoichiometric Variation

In a ternary system such as ABZ, the stoichiometric variation within each material is wide, which opens the possibility for a multitude of compositions for functionalization. For instance, with different metal to chalcogen ratios, sodium antimony sulfides occur in two different compositions, i.e., NaSbS_2_ and Na_3_SbS_4_. NaSbS_2_ has a direct band gap in the range of 1.4–1.73 eV depending on the polymorph, as shown in Figure 3, whereas tetragonal Na_3_SbS_4_ has been reported to have a theoretical band gap of 1.67 eV.^41^ The band gaps for both compositions are in a similar range, a range which is suitable for photovoltaics. However, Na_3_SbS_4_ has shown more promise in the field of energy storage given its superior ionic conductivity. Cubic and tetragonal Na_3_SbS_4_ have been researched for solid electrolytes in Na-ion batteries, and the phases show ionic conductivities of 3 and 1.1 mS cm^–1^ respectively.^40,42^ These ionic conductivities are three orders of magnitude higher compared to that of the analog cubic NaSbS_2_. This difference in the conductivity is allied with the difference in the density of states for NaSbS_2_ and Na_3_SbS_4_. The upper valence bands of both materials differ in their interactions with the lone pair in the Sb 5s orbital. NaSbS_2_ shows interactions between the S 3p orbital and Sb lone-pair electrons, while in Na_3_SbS_4_ this contribution is not present and the upper valence band is mostly made of S p orbitals. In Na_3_SbS_4_ the high ionic conductivity can be attributed to the Na-ion diffusion pathways in the crystal framework, which act as 3D tunnels for ions and are formed by the two different types of sodium–sulfur polyhedra.^41,42^ Similar to the example above, targeted functionalities can be induced by tuning the metal to chalcogen ratios in the material design for various alkali metal-based chalcogenide systems. CsCu_4_Se_3_ is limited to a ZT of 0.06 due to a low Seebeck coefficient, unlike CsCu_5_Se_3_ with a ZT of 1.81; the change in composition gives a huge increase in terms of thermoelectric properties.^44,47^ However, one must consider that many of these phases of different material compositions would be metastable in nature, and intelligent synthetic routes with detailed understanding of the underlying chemistry will be needed for materializing such compositions.

## 3 Engineering Compositions

Compositional tunability is one of the main traits of metal chalcogenides. Generally, metal chalcogenides are capable of adapting the modulations in their structural framework while being exposed to compositional variation or metal to chalcogen stoichiometric modifications. Historically, copper-based chalcogenides have exhibited the tendency to generate numerous stoichiometric variations and their corresponding crystal structures.^60^ Furthermore, these stoichiometric structures can be investigated in terms of cation exchange in order to introduce additional elements into the system.^61^ The aforementioned trend can also be extended to ternary alkali metal-based chalcogenides. For ABZ, substitution at all three sites A, B and Z is possible; this can lead to a plethora of possible compositions with a wide range of applications. The variety of substitution at different positions in the ABZ structure has a significant influence on electrical and physical material characteristics. Typically, cations and anions with similar atomic radii exhibit substitution in corresponding sites within the stoichiometric composition. For instance, Na can replace K, while Se can replace S or Te, resulting in the formation of noncentrosymmetric structures. In the context of metal sites, it is possible to substitute metals with similar valence states or ionic radii at the designated metal (B) site. Iyer et al. and our group have demonstrated this phenomenon through the substitution of Sb in place of As and Bi sites in NaAsSe_2_ and NaBiS_2_, respectively.^19,62^

Modifications in the structural composition of materials or the arrangement of their constituent atoms possess a propensity to elicit significant transformations in a material’s inherent characteristics. In the case of alterations in the metal site, the structure causes a substantial mass variation in the lattice. These oscillations have a significant impact on the thermal and charge transport characteristics of these materials and will be useful in controlling the overall performance. The doping studies on alkali metal chalcogenides have shown that the figure of merit of ZT can be substantially improved, mainly by raising the power factor. For example, our group showed that the thermoelectric transport characteristics of NaBiS_2_, upon Sb substitution, exhibit a remarkably low thermal conductivity and n-type transport behavior.^19^ Furthermore, the introduction of external cations, such as antimony, onto the bismuth sites results in increased configuration entropy and point defects, which effectively scatter phonons and reduce thermal conductivity.

The use of composition engineering could modify a direct-band gap semiconductor (i.e., a semiconductor that exhibits light absorption) into an indirect-band gap material and transform a topological insulator into an ordinary insulator. A computational study conducted systematically demonstrated that manipulating Li doping levels can effectively regulate the band gap of NaSbS_2_. By means of controlled substitution of Li, a band gap within the range of 0.6–1.7 eV has been attained.^63^ The process of substituting elements in ternary metal chalcogenides has the potential to induce changes in their properties and trigger a transition from the thermodynamically stable crystal phase to the stabilized metastable phase. For ternary alkali metal chalcogenides, most of the noncentrosymmetric phases convert to centrosymmetric stabilized phases. In certain instances, noncentrosymmetric phases can be stabilized through alterations in synthetic conditions. Additionally, specific alkali metals have been found to facilitate the stabilization of a wide range of chain conformers. In this context, the substitution of transition metals or different chalcogenides was found to stabilize the noncentrosymmetric β- and γ-phases of LiAsSe_2_.^62,64^ Additionally, the noncentrosymmetric phases were stabilized by the substitution of Sb and Na in NaAsSe_2_ and KAsZ_2_ (Z = S, Se), respectively.^62,65^ The estimation of physical properties, such as the melting point, is of the utmost importance in nonlinear optics (NLO). The incorporation of larger anionic substitutes has been observed to reduce the melting point of materials by increasing the length of metal-to-anion bonds, thereby lowering the stability of the materials and, consequently, their melting point.

The introduction of a third metal, whether from the main group or transition elements, to the ternary alkali metal results in the formation of a vast array of quaternary chalcogenides denoted as ABB′Z. These compounds demonstrate a diverse array of chemical characteristics that encompass the entirety of the periodic table. Theoretical and computational advances have led to the discovery of a vast array of thermodynamically stable quaternary chalcogenides, thereby expediting their synthesis in the solid state. A few representative examples of this class of compounds are ACuB′’S (A = K, Na; B′ = Bi, Sb) and ABB′Z_4_ (A = K, Rb, Cs, Tl; B = Ga, In; B= Ge, Sn; Z = S, Se).^66,67^ In this Perspective, the decision to restrict our compositions solely to ternary chalcogenides is based on the vast number of potential compositions that arise when considering quaternary chalcogenides.

## 4 Synthesis Approach

Modern chemistry represents a vast and multifaceted domain of scientific research wherein diverse subdisciplines synergistically converge to propel our fundamental understanding of the characteristics of novel functional materials and the means by which they may be effectively manipulated. With respect to that, the fundamental objective of chemistry is to ascertain and advance systematic and logical methodologies for transforming the art of synthesis into a scientific process. The quest for novel functional materials and effective synthetic methods is a fundamental endeavor in the field of chemistry. One notable trend in this area of study pertains to the integration of transition or main group metals into alkali chalcogenides, which enhances the structural variability and characteristics of the chalcogenides. Considering the extensive range of molecular constituents available for alkali metal chalcogenides and the coordination chemistry exhibited by diverse transition and main group metals (Bi, Sb, Fe, Cu, Zn, Ag, etc.), it is pertinent to explore their potential.

Over the years, the formation of alkali metal chalcogenides with exciting new structures necessitated the use of solid reactants, which were exposed to extremely high temperatures (>800 °C) and extended reaction periods from 1 to 8 days. The rationale behind this approach is the sluggish diffusion of reactants that is anticipated in the absence of a solvent. This methodology has been employed to synthesize a considerable number of alkali metal chalcogenides that are currently known (Table 1 and Figure 2). Undoubtedly, high-temperature techniques have exhibited remarkable efficacy in this regard. Nevertheless, the reaction conditions employed in these methods tend to promote the structures and compositions that are the thermodynamically most stable. The diversity of compounds that can be obtained is restricted due to the influence of temperature, as lower temperatures can stabilize phases that may not persist at higher temperatures. As compared with solid-state synthesis, the methodologies for synthesizing molecular systems at relatively lower temperatures have yielded an abundance of novel coordination compounds, organic molecules, clusters, and complexes.

**Table 1:** Various Synthesis Methods of ABZ Materials

material	synthesis method	crystal structure	synthesis conditions	band gap
CsAg_5_Te_3_^48^	salt melt	tetragonal	800 °C for 2 h	0.67 eV
KCu_4_S_3_^49^	salt melt	tetragonal	800 °C for 1 h	n/a
LiBi_3_S_5_^50^	salt melt	monoclinic	600 °C for 4 h	n/a
KBi_3_S_5_^51^	solid state flux	orthorhombic	300 °C for 5 days	1.21 eV
CsBiS_2_^52^	solid state flux	monoclinic	290 °C for ∼5 days	1.43 eV optical band gap
		rhombohedral	350 °C for ∼7 days	1.11 eV optical band gap
NaFeS_2_^53^	hydrothermal	monoclinic	180 °C for 24 h	2.01 eV
NaCu_5_S_3_^54^	hydrothermal	hexagonal	140 °C for 5days	n/a
KCu_7_S_4_^55^	hydrothermal	tetragonal	150–200 °C for 24 h	1.69–1.74 eV
KFeS_2_and KFe_2_S_3_^56^	solvothermal	monoclinic (KFeS_2_)	190 °C for 18–48h	n/a
		orthorhombic (KFe_2_S_3_)		
NaInS_2_^57^	solvothermal	hexagonal	180 °C for 12 h	n/a
KInS_2_^57^	solvothermal	monoclinic	180 °C for 12 h	n/a
CsCu_5_S_3_^58^	colloidal synthesis	orthorhombic	260 °C for 15 min	1.4 eV
NaBiZ_2_(Z = S, Se)^59^	colloidal synthesis	rock salt	180 °C for 3 h	direct: 1.4–1.45 eV
				indirect: 1–1.1 eV
ABZ_2_(A= Li, Na; B= Bi, Sb; Z = S, Se)^35^	colloidal synthesis	rock salt	200–300 °C for 1–30 min	direct: 1.4–1.51 eV
				indirect:1.52–1.67 eV
NaBi_1–*x*_Sb_*x*_Se_2_	colloidal synthesis	rock salt	200–240 °C for 30–40 min	n/a
NaBiSe_2–*y*_S_*y*_^19^				

The molten salt (flux growth) technique is a noteworthy approach in ABZ synthesis, wherein strongly polarizing agents such as molten salt or molecular solvents are employed to facilitate the process at lower temperatures ranging from 100 to 600 °C. In contrast to high-temperature synthesis, the utilization of less severe thermal conditions in this synthetic technique facilitates the formation of metastable phases that may not be attainable under high-temperature conditions. The utilization of the flux method results in a reduction of the reaction temperature due to its ability to facilitate rapid mass transfer transport within the liquid phase through convection and diffusion mechanisms. The group of Kanatzidis developed a remarkable number of novel structures for alkali metal polychalcogenides utilizing the technique of molten flux synthesis.^68^ Undoubtedly, the molten salt method has been employed for the production of numerous alkali metal chalcogenides (Table 1). However, there is still a significant amount of fundamental chemical knowledge to be uncovered regarding materials synthesis through this method. This is due to the unique nature of the process and the chemistry involved, which differ significantly from traditional wet-chemistry methods. The unambiguous understanding of the molecular-level structure of ionic salt species in numerous systems remains elusive.^69^

In recent decades, the solvothermal/hydrothermal method has gained attention for synthesizing ternary alkali metal chalcogenides. Analogous to the molten salt methodology, this technique operates at lower reaction temperatures, thereby enabling the synthesis of metastable phases along with the kinetic control and mechanistic study of ternary chalcogenides. Schafer and associates conducted a pioneering investigation in the field. They synthesized a range of alkali metal chalcogenides by dissolving Sb_2_S_3_ in aqueous A,S/ASH solutions (where A represents Na, K, Rb, and Cs) at temperatures ranging from 120 to 180 °C.^70^ A large number of ternary alkali chalcogenides have been synthesized through hydrothermal or solvothermal methods (Table 1). In contrast to solid-state reactions, solution-based synthetic techniques such as the solvothermal approach have been shown to offer novel reaction and thermochemical pathways and facile mild-chemistry procedures that can yield the desired morphologies, dimensions, and compositions.

**Figure 4:**
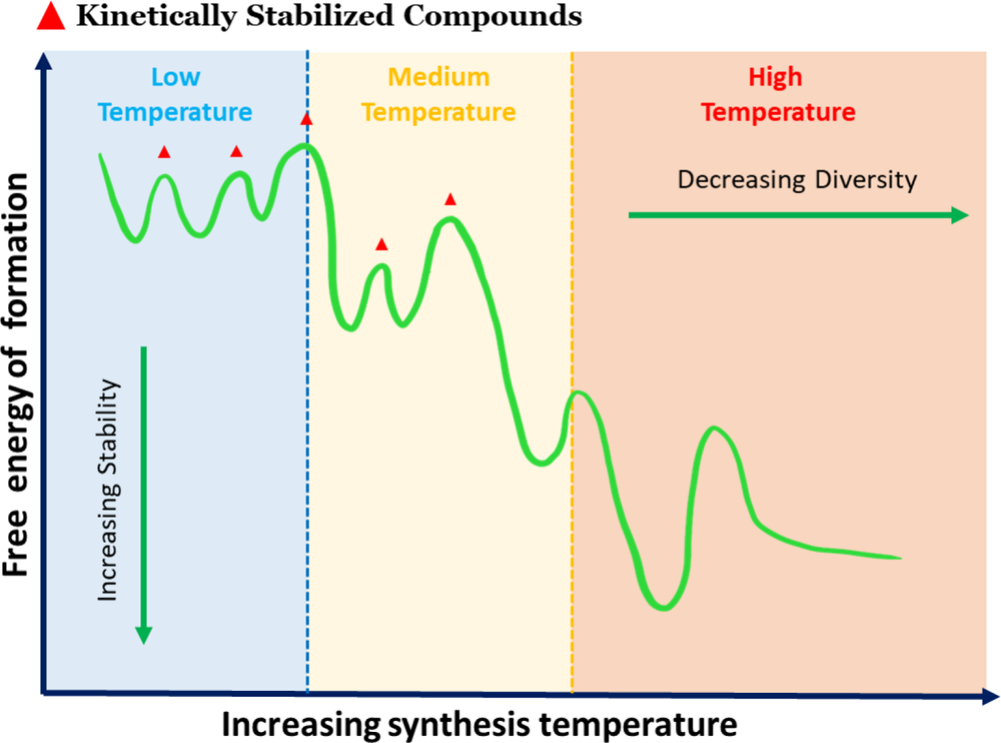
Schematic illustration of different phases that are stabilized in different temperature regimes. As temperature increases, the potential number of compounds decreases. Adapted with permission from ref (68). Copyright 2017 American Chemical Society.

Figure 4 provides a conceptual representation of the complex relationship between temperature and the ability to effectively stabilize a gradual broader array of compounds within a specific reaction system. The likelihood of the formation of metastable phases relative to thermodynamically stable phases diminishes as the reaction temperature increases. In the realm of ABZ compounds, the majority of experimental validation predominantly focuses on thermodynamically stable phases. This preference arises from the prevalent utilization of high-temperature synthetic techniques, which precludes the formation of metastable phases. The generation of metastable phases in ABZ compounds requires a reduction in the reaction temperature, which in turn demands meticulous manipulation of precursor chemistry and delicate regulation of reaction conditions. This is primarily due to the inherent disparity in reactivity between A (alkali metal) and B (transition or main group metal).

Although these compounds have been extensively studied and established for diverse applications pertaining to energy conversion and storage. The exceptional properties of these materials have yet to be thoroughly investigated in the nanoscale regime. Moreover, the mechanistic understanding of the formation of these functional materials has not been extensively explored. Nanoscale materials are known to possess distinctive physical and chemical properties, which might contribute to improvements in engineered materials. These enhancements may include superior magnetic properties and enhanced electrical and optical activity. Furthermore, the amplified reactivity of these materials can be attributed to their increased surface area in comparison to that of their bulk material equivalents. The unique characteristics of nanoscale materials offer them the potential to enhance the efficacy of a diverse array of applications. Additionally, the colloidal hot injection approach has been the centerpiece in the synthesis of nanomaterials after its first introduction in the last century, as it not only provides fine control over many parameters like size, shape, and composition but also provides mechanistic insights for the reaction mechanism. In a recent study focused on the colloidal approach for ternary alkali metal chalcogenides, Vela and his colleagues successfully synthesized NaBZ_2_.^59^ This study represents one of the few and first colloidal synthetic approaches to this topic. In a further advancement to this study, a generalized synthetic protocol was developed for NaBZ_2_ (B= Bi, Sb; Z= S, Se).^71^ The present study involves an analysis of surface chemistry, a factor that was deemed neither significant nor viable in any of the previously mentioned synthesis methodologies.

The role of surface chemistry is of paramount importance in both the synthesis and application processes.^11,20,72^ The fundamental attribute of nanomaterials resides in their extraordinary surface-to-volume ratio, which is of paramount significance to their surfaces by dictating their physical and chemical properties. Surface ligands play a pivotal role not only in the realm of synthesis but also in the intricate domains of processing and application. Surface engineering enables us to effectively manipulate or coat the surfaces of nanomaterials, thereby augmenting their inherent characteristics and overall performance. The proliferation of research instruments capable of manipulating material surfaces while preserving the fundamental properties of the underlying core material has ushered in a novel realm of investigation within the field of surface engineering. In this particular realm, the utilization of ligand exchange methodologies has fortuitously revealed the profound influence of surface chemistry on the electronic structure. Undoubtedly, the implementation of postsynthesis surface chemistry modification has demonstrated its capacity to engender advantageous attributes that prove invaluable in the realms of processing and application.

## 5 Properties and Opportunities

The extensive compositional space of alkali metal-based ternary chalcogenides has immense potential in a range of energy and optoelectronic applications. High polarizability, weaker interatomic bonds, high stability, low thermal conductivity, and charge transport collectively create opportunities for the exploration of these materials for technological applications. Most of these materials display layered structures with embedded properties like photocurrent response,^73^ magnetism,^74^ exfoliation,^75^ and lithium-ion conductivity.^76^ Given such striking features of the ABZ materials, we further outlined our prospect on some of the properties and opportunities, which are briefly explained in this section.

One key property that has been explored in many ABZ material compositions is the low thermal conductivity. They intrinsically possess low thermal conductivity, which means they have a reduced ability to transfer heat. Thanks to the vast composition engineering, these materials can regulate and manipulate thermal properties for innovative thermal management strategies in energy conversion. This has been of special interest for technologies in which thermal insulation or heat dissipation is required. For instance, thermoelectric devices perform the direct conversion of thermal energy into electricity from various forms of heat sources, including waste heat. Typically, the performance of these devices is determined through a graphical plot of merit, ZT. Although, the efficiency of thermoelectric materials can be regulated through various strategies like band engineering,^77^ resonant states, energy barrier filtering^78^ and large-scale nano structuring,^79^ it is still critical to develop materials at low cost with a high conversion efficiency for the wide societal utilization of thermoelectric technology. Incorporation of ABZ has notable potential, given their attractive features of low cost, diverse structures, high electrical conductivity, and low thermal conductivity.

**Figure 5:**
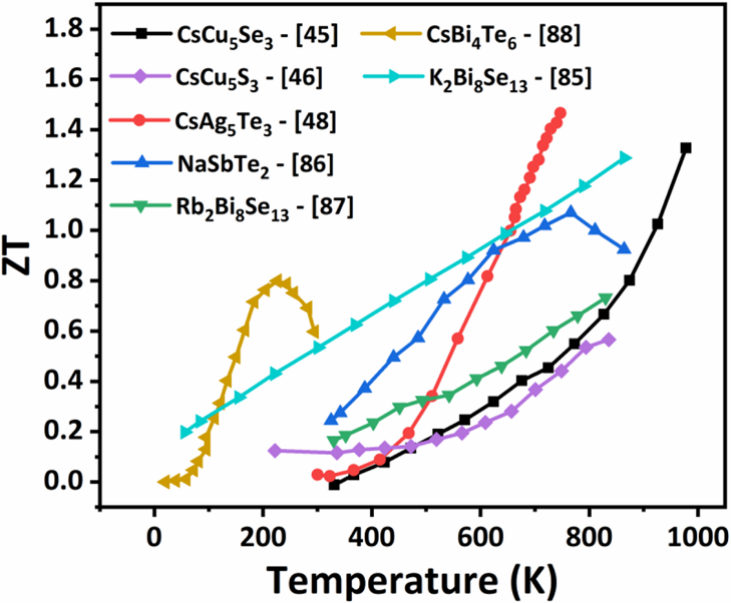
Thermoelectric performance of various ABZ materials.^45,46,48,85−88^

Currently, PbTe-based compounds have been the leading materials with an intermediate temperature regime (∼800 K) integrated into commercial devices. However, the performance of PbTe is maximized to ∼900 K, which is too low over the temperatures of interest for most of the potential commercial applications. As a solution, various ABZ materials could be promising midtemperature materials with high merit values, as they inhibit the low thermal conductive property intrinsically when compared to other conventional materials. Table 2 delivers an ideal overview of thermal conductivities of ABZ materials and other conventional materials. Some of the notable thermoelectric performances of ABZ materials in different temperature regimes reported so far from the research community are represented in Figure 5. For instance, CsAg_5_Te_3_ is a p-type thermoelectric material with an ultralow thermal conductivity exhibiting a high figure merit of 1.5 at 727 k as reported by Hua et.al. in a single-phase compound.^48^ The prospect of nanostructuring is commonly employed to enhance the thermoelectric property in conventional thermoelectric materials. However, compositions such as NaPb_*m*_SbTe_*m*+2_ can achieve a very high performance *ZT*_avg_ of 1.1 over 323–673 K without nanostructuring. This feasibility can simplify the fabrication process and reduce the production cost of commercial devices.

**Table 2:** Thermal Conductivity Comparison between Conventional Materials and ABZ Materials

composition	thermal conductivity
conventional materials	
Bi_2_Te_3_^80^	0.37 W·m^–1^·K^–1^
PbSe^81^	1.53 W·m^–1^·K^–1^
PbS^82^	0.4 W·m^–1^·K^–1^
SnSe^83^	0.23 W·m^–1^·K^–1^
SnS^84^	0.4 W·m^–1^·K^–1^
ABZ materials	
CsAg_5_Te_3_^48^	0.18 W·m^–1^·K^–1^
K_2_Bi_8_Se_13_^85^	0.20 W·m^–1^·K^–1^
CsCu_5_Se_3_^45^	0.40 W·m^–1^·K^–1^
CsCu_5_S_3_^46^	0.56 W·m^–1^·K^–1^
NaSbTe^86^	0.7 W·m^–1^·K^–1^
Rb_2_Bi_8_Se_13_^87^	0.46 W·m^–1^·K^–1^

Excellent chemical stability and mechanical integrity, which are crucial from a practicality viewpoint, are found in NaPb_*m*_SbTe_*m*+2_ compounds. These materials retain their thermoelectric performance even under harsh operating conditions and extended exposure to elevated temperatures. This resilience highlights their potential for long-term and reliable performance in real-world applications. Importantly, the exceptional properties of these solid solutions offer numerous applications in energy conversion and harvesting technologies. They can be employed in waste heat recovery systems to enhance energy efficiency in industrial processes and power generation. Additionally, these materials can be integrated into thermoelectric devices for on-chip cooling, thermal sensors, and other electronic applications that require efficient heat management.

Another property of high relevance from a technological viewpoint is the optoelectronic behavior of ABZ materials. It is a discipline that pertains to the manipulation and regulation of light and electrical signals. This field encompasses a range of devices, including photodetectors, solar cells, light-emitting diodes (LEDs), and lasers. Overall, the key features of ABZ including their wide bandgaps, high optical absorption, efficient charge transport, tunable energy levels, stability, compatibility, solution processability, versatility, and cost-effectiveness make them highly promising materials for nonlinear optics, optical sensing, optical coatings, and integrated photonics. One such example is NaSbS_2_, which has large absorption coefficients (10^–4^ to 10^–5^ cm^–1^) in the visible region and in the range of 1.5 to 1.8 eV.^89^ Taking an example of the versatility of ABZ materials in optoelectronic studies, Iyer et.al highlights the potential applications of Na_1–*x*_K_*x*_AsQ_2_ in optoelectronic devices.^65^ The strong SHG response, along with the tunable structure and high optical stability, makes Na_1–*x*_K_*x*_AsQ_2_ a promising material for various applications. For example, in optical communications, the material’s nonlinear optical properties are exploited for signal processing and data transmission. Moreover, Na_1–*x*_K_*x*_AsQ_2_ can also be a potential material for photonic devices, such as optical modulators and sensors, where its tunable structure allows for tailoring the material’s performance to specific device requirements.

Furthermore, a stable polar structure is a key feature that brings unique optical properties to existence. The stability of the materials is crucial for ensuring the reliability and long-term performance of nonlinear optical devices. However, stabilizing a polar structure is a great challenge for the research community. The ABZ materials AGa_5_S_8_^90^ and γ-NaAsSe_2_^91^ are thermodynamically stable in different environmental harsh conditions. Their high nonlinearity, combined with structural and thermal stability, makes them promising candidates for infrared region frequency conversion devices. However, continued research and development in this field are needed to enhance our understanding and unlock ABZ materials as potential candidates in advanced nonlinear optical devices.

**Figure 6:**
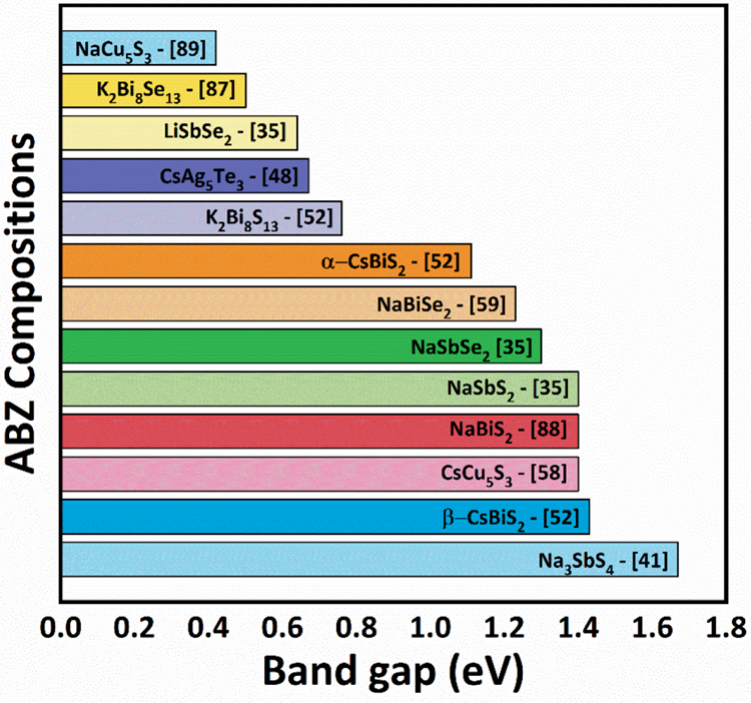
Experimentally validated bandgaps of ABZ materials reported in the literature.^35,41,48,52,58,59,92−94^

Tuning absorption spectra is necessary for a wide range of applications to align with the solar spectrum to maximize the light harvesting and energy conversion efficiently. Figure 6 shows the range of band gaps of the experimentally synthesized ABZ compositions. The wide window of 0.3–1.8 eV serves as a linchpin for the optical diversity of the future ABZ compositions that yet has to be materialized experimentally.

In ascertaining the electronic configuration, optical characteristics, and general conduct of a substance, the charge transport phenomenon plays a pivotal role. The phenomenon of charge transfer in alkali metal chalcogenides is commonly understood as the exchange of electrons between the alkali metal cations (e.g., Li^+^, Na^+^, and K^+^) and chalcogen anions (e.g., S^2–^, Se^2–^, and Te^2–^). The charge carrier dynamics and trap states in ABZ materials are expected to be similar to those of other classes of metal chalcogenides such as II–VI materials. However, the high compositional tunability of ABZ materials gives rise to unique electronic behavior. For instance, semi-insulating behavior with a high resistivity and low concentration of free charge carriers in Cs_2_Hg_6_S_7_ has been observed.^95^ The dynamics of charge carriers in Cs_2_Hg_6_S_7_ were investigated using photoinduced current transient spectroscopy measurements. It was revealed that Cs_2_Hg_6_S_7_ demonstrates extended carrier lifetimes, which suggests a reduced rate of recombination. Another excellent example of ABZ materials with high hole mobility is NaCu_4_Se_4,_ which shows potential for being integrated in electronic devices and high-speed electronics, where efficient charge transport is crucial.^96^

Though minimal trap densities are the crucial factors of charge transport, the general findings suggest that nanocrystals exhibit a low density of trapping states, which decrease the loss of charge carriers upon trapping and recombination. Yang et al. reported KBiS_2_ nanocrystals with high carrier mobility and low trap density that enable the efficient detection of light signals, resulting in enhanced sensitivity and response times.^97^ Photodetectors based on alkali metal bismuth chalcogenide nanocrystals offer potential applications in imaging systems, optical communications, and sensing devices where high-performance and reliable light detection are essential. On the other side, the abundance of defects at the interface and grain boundaries in perovskites caused by their ionic nature often negatively impacts the performance of perovskite solar cells. Employing ABZ materials as an interlayer between the perovskite film and hole transport layer in solar cells can enhance the power conversion efficiency. In brief, the energy level alignments of the ABZ material between the perovskite and hole transport layer strongly suppress the nonradiative recombination in solar cells. Recently, CsCu_5_S_3_ nanocrystals are utilized as interlayers and delivered a champion power conversion efficiency (PCE) of 22.29% with good reproducibility and stability.^58^ The impressive efficiency achieved from these ABZ materials paves the way further in advancing the development of efficient and cost-effective solar energy conversion technologies.

The added benefits of band structure engineering in the ternary ABZ composition, photogenerated carrier separation, and chemical stability have opened up a new direction toward their use as photocatalysts. However, the operational stability and reusability of the chalcogenides, specifically sulfides, are limiting factors when looking from a technoeconomic perspective. Nevertheless, a few recent works on the photocatalytic properties of ABZ materials are instrumental for the longevity of this nascent field. For instance, NaFeS_2_ has been reported to be a high performance electrocatalytic material for redox reactions.^53^ Some of the suitable bandgaps of ABZ materials mentioned in Figure 6 are more advantageous in allowing absorption of visible light, abundant in the solar spectrum. For example, nanostructured NaBiS_2_ is employed as a visible-range photocatalyst to degrade 2,4-dichlorophenol, a toxic industrial pollutant. Such promising photocatalytic materials are important for environmental remediation and wastewater treatment.^98^

Another promising platform driven by ABZ materials is efficient and sustainable energy storage. Generally, chemical energy storage devices are known as batteries and are fundamental technological components in a variety of energy storage systems. Batteries have been a popular technology option for numerous applications. Nevertheless, contemporary battery technologies are subject to cost restrictions and performance limitations, including short shelf life and cycle life, as well as slow charging and discharging rates at high-power densities.^99^ Some of the key components in batteries can greatly affect the performance of batteries, mainly anodes in lithium-ion batteries (LIBs). However, many compositions of anode materials with high theoretical capacity have been developed, such as alloy type (Si, Sn, P, Ge, etc.),^100−103^ conversion type (Fe_3_O_4_, MnO, Mn_3_O_4_, FeS, FeS_2_, NaV_2_O_5_, CoNiO_2_, etc.),^104−110^ and conversion–alloy type (Cu_3_Ge, Li_3_P, etc.).^111,112^ However, the limited rate performance and charge–discharge drawbacks pushed researchers to focus on alternate materials. In the class of ABZ materials, compositions such as LiInSe_2_ and NaFeS_2_ can achieve a high lithium storage capacity, which is essential for improving the energy density of lithium-ion batteries.^113,114^ These materials exhibit reversible lithium insertion and extraction, allowing for efficient energy storage and release during charge–discharge cycles. Another unique feature of these materials to take into account is their cycling stability. In all, ABZ materials are considered a new class of potential anode materials for LIBs (lithium-ion batteries), PIBs (potassium-ion batteries), and SIBs (sodium-ion batteries). However, a recent study on NaFeS_2_ as new anode material in a Li-ion battery reported by Zhang et al detailed a higher capacity, longer cycle life (1157 mAh/g after 500 cycles), and better rate performance (618 mAh/g at 5 A·g–1).^114^ However, the theoretical study validates the metallic conductivity in NaFeS_2_, which is prone to higher electron transfer rates. Even more fascinatingly, NaFeS_2_ exhibits promising potential in PIBs and SIBs. In the NaFeS_2_/Na system, a specific capacity of 442 mAh/g initially retains 254 mAh/g after 1000 cycles at a current density of 300 mA/g. Moreover, in the NaFeS_2_/K system, it achieves a capacity of 241 mAh/g, which increases to 265 mAh/g after 450 cycles at 100 mA/g.^114^ This excellent cycling stability exhibited by ABZ promises long-term performance and durability of the battery, which is crucial for practical applications and contributes to the overall reliability of the energy storage system.

Importantly, the abundant constituent elements in the ABZ compositions such as NaBiS_2_, Na_0.5_NbS_2_, LiInSe_2_, and NaFeS_2,_ ensures a readily available and cost-effective supply, avoiding the reliance on rare earth and expensive materials.^113−116^ Promoting earth-abundant alkali metal chalcogenides in batteries encourages more accessible and environmentally conscious energy storage technologies.

## Conclusions and Considerations for the Future

In this Perspective, we have introduced a library of ternary compositions based on alkali metal chalcogenides. However, the existing collection of these ternary ABZ compounds still lacks many possible combinations. This is why so much work remains in this compositional landscape to gain better fundamental insight into how compositional engineering can relate to structure–property relationships. We have discussed the flexibility of the crystal structures of this compositional landscape. The tunability of electronic band structure within a given elemental combination by stoichiometric variation opens many interesting avenues of study wherein the insights from theoretical and computational studies can direct future material design.

This Perspective discussed the advancement in composition engineering in these materials, where doping/alloying of a third metal on either A or B sites can accentuate the existing properties of the pristine material composition. Given the vast library of ABZ combinations, composition engineering via doping/alloying in this material class might bring a 10-fold increase in the number of viable material sets. One must be careful, as this will enormously increase the efforts to construct meaningful structure–property relationships in ABZ compounds. The reason behind this is the vast phase diagrams that these compositions exhibit. Given the structural flexibility of metal chalcogenide lattice frameworks, multiple unique polymorphs can be synthesized with unique properties. These different polymorphs when subjected to doping or alloying can either yield metastable structures with technologically useful properties or could yield hypothetical compounds with higher energy than the lowest-energy crystal structures that they are derived from.

The optoelectronic and thermoelectric properties of ABZ compounds are also discussed herein. Real-time analysis of these functional energy materials is required using sophisticated operando spectroscopy and microscopy techniques. The knowledge on defect control and engineering in ABZ compositions is very scarce, and a consistent growth in this area will be prerequisite for establishing their candidacy as functional materials for device applications. Further, surface functionalization and engineering through the use of composites are another possibility to harness the best performance for each individual application.

We also discuss the synthetic approaches being employed over the years to materialize the known and realizable ABZ compositions. Most of the compositions realized so far are being formed under a high-temperature synthetic regime. This might be due to the under-reactivity issues of the common metal precursors. However, this precludes us from accessing the phases that are metastable in nature at lower temperatures. These metastable phases either can show phase transition toward the thermodynamically stable phase or can decompose to form mixed phases when heated at high temperatures. Given this, a very interesting avenue to explore is the use of solution-based approaches for synthesizing ABZ compositions, for instance, solvothermal or hydrothermal approaches. In our own experience in exploring the 1T′ metastable phase synthesis of transition metal chalcogenides, we have found that colloidal synthesis gives better control in phase engineering in materials such as MoS_2_ and WS_2_ compared to the chemical exfoliation approach.^13,117,118^ In colloidal synthesis, molecular precursors and organic surfactants/ligands are heated at high temperatures, where precursors decompose to form monomers, which then nucleate and grow as NCs. Colloidal synthesis chemistry offers exquisite control over the size, shape, and composition of crystals through the precursor–ligand–temperature interplay. It has the capability to access metastable phases and to precisely control the nature and extent of interfaces between different crystal domains.

One added key advantage of employing colloidal synthetic approaches is the ability to synthesize the materials in nanoscale dimensions. Reducing the crystal size to the nanoscale regime extends the range of unique properties that can be relevant from a technological viewpoint. Quantum confinement and tunable photoluminescence can be taken as good examples of nanoscale-regime phenomena. These additional optoelectronic properties could be highly beneficial when considering the candidacy of ABZ compositions for optical devices. Moreover, nanocrystals can be explored as building blocks to form dense superstructure solids that cannot be produced with traditional methods. Given that the main applications of ABZ compounds as highlighted in this Perspective are photovoltaics and thermoelectric materials, solution processability of nanoinks would be advantageous for next-generation flexible device fabrications. Nevertheless, fine synthetic control over the morphology, dimensionality, and surface structuring at the nanoscale represents one of the final milestones in material design that will give access to new unique properties exclusive to nanoregime and breakthrough performance in photovoltaics and catalysis, among others.

To this end, we emphasize that the synthesis of an unknown composition is challenging and complex due to the dependence of synthesizability on a multitude of interconnected parameters such as reaction temperature, pressure, reactivity of precursors, and crystallization kinetics. The discovery, design, and application of a new composition landscape are only possible with continual communication between theoreticians and experimentalists. A fundamental understanding of functionality descriptors (e.g., band gaps and electronic structure, electric or thermal conductivities) in combination with synthesis descriptors (e.g., formation enthalpies, temperature-dependent free energies) will immensely benefit experimentalists in synthesizing novel compositions and validating the theory-identified properties.
